# Pancreatic mucinous cystic neoplasms in a young female: A case report

**DOI:** 10.1097/MD.0000000000049853

**Published:** 2026-07-24

**Authors:** Ethan Burg, Gabrielle K. Sharbin, Max E. Edeson, Hossein Rabiei Samani, Hanieh K. Hosseini, Davood K. Hosseini, Shil Patel, Jonathan D. Weinberger, Hongfa Zhu, Rosario Ligresti

**Affiliations:** a Department of Gastroenterology, Hackensack Meridian School of Medicine, Hackensack University Medical Center, Hackensack, NJ; b Department of Medicine, Geisel School of Medicine at Dartmouth, Dartmouth Hitchcock Medical Center, Lebanon, NH; c Department of Medicine, Albert Einstein College of Medicine, Montefiore Medical Center, Bronx, NY; d Department of Medicine, Icahn School of Medicine at Mount Sinai, Mount Sinai Morningside/West, New York, NY; e Retzky College of Pharmacy University of Illinois, Chicago, IL; f Thomas J. Long School of Pharmacy, Stockton, CA; g Department of Gastroenterology, Palisades Medical Center, Hackensack, NJ; h Department of Pathology, Hackensack University Medical Center, Hackensack, NJ.

**Keywords:** cystic mass, mucinous cystic neoplasms, pancreatic cystic neoplasms

## Abstract

**Rationale:**

Pancreatic cystic neoplasms (PCNs) encompass a wide spectrum of disease from benign to malignant. Mucinous cystic neoplasms (MCNs) are the second most common PCNs, typically occurring as solitary masses in the pancreatic body or tail of middle-aged women. They are characterized by elevated cyst fluid carcinoembryonic antigen (CEA) and low amylase due to lack of ductal communication. While MCNs have malignant potential, and lesions >3 cm are higher risk, size alone is not an absolute predictor of malignancy. We present a rare case of an exceptionally large, yet benign, PCN in a 24-year-old female, with a paradoxical cyst fluid biomarker profile.

**Patient concerns:**

A 24-year-old female with a known pancreatic tail cystic mass presented with acute-on-chronic abdominal pain, nausea, and vomiting – endorsing epigastric pain for the past year. The lesion was initially identified in January 2024 on imaging, measuring 11.7 × 10.2 × 8.1 cm, with prior non-diagnostic cytology.

**Diagnoses:**

Abdominal magnetic resonance imaging with cholangiopancreatography demonstrated a complex cystic mass measuring 8.4 × 12.8 × 16 cm abutting the pancreas with moderate volume ascites. Endoscopic ultrasound (EUS)-guided fine-needle biopsy revealed a 12 cm complex mass encompassing the distal pancreas. Surgical pathology confirmed a noninvasive, low-grade MCN with no evidence of high-grade dysplasia.

**Interventions:**

Given the significant symptom burden and cyst size, she underwent distal pancreatectomy with splenectomy; the cystic mass was adherent to the splenic vessels at the hilum, precluding a spleen-sparing procedure.

**Outcomes:**

Pathology revealed a 14 × 11 × 7 cm multilocular cystic mass consistent with a benign, noninvasive, low-grade MCN. The patient had an uncomplicated post-operative course and is following with outpatient providers.

**Lessons:**

This case highlights the limitations of cyst size as a predictor of malignancy in MCN. Further, there is a possibility of discordant cyst fluid biomarkers such as low CEA and high amylase as opposed to the inverse, as seen here, which may reflect sample heterogeneity, partial ductal communication, or mixed lesions. This case therefore emphasizes the importance of integrating imaging, cyst fluid biomarkers, histopathology, and molecular testing together for accurate classification and individualized management of PCNs.

## 1. Introduction

Pancreatic cystic neoplasms (PCNs) encompass a wide range of pathologies and presentations.^[1]^ Early distinction between the various types of PCNs is vital in clinical management.^[1–3]^ Mucinous cystic neoplasms (MCNs) are the second most common PCNs.^[2–4]^ They typically occur as solitary masses in the pancreatic body or tail,^[2,3]^ and are found almost exclusively in middle-aged women.^[2,3,5,6]^ MCNs are mucin-producing tumors and, while the majority are benign, they have the ability to undergo neoplastic transformation.^[2,3]^ Features worrisome for malignancy include elevated serum carbohydrate antigen 19-9 (CA 19-9), the presence of enhancing mural nodules ≥5 mm, peripheral eggshell calcifications, pancreatic duct dilation, lesion size >3 cm, or lesion growth rate ≥2.5 mm per year.^[2–4]^

Cyst fluid analysis for MCNs usually shows elevated carcinoembryonic antigen (CEA) and low amylase, due to lack of ductal communication.^[3,7]^ However, discordant biomarker profiles can be present and complicate diagnosis, raising consideration for alternate processes.

Here we present a case of a young female with an exceptionally large (16 cm) MCN, which, despite the presence of elevated tumor serum markers and large lesion size, was ultimately found to be benign.

## 2. Case report

**Figure 1.**
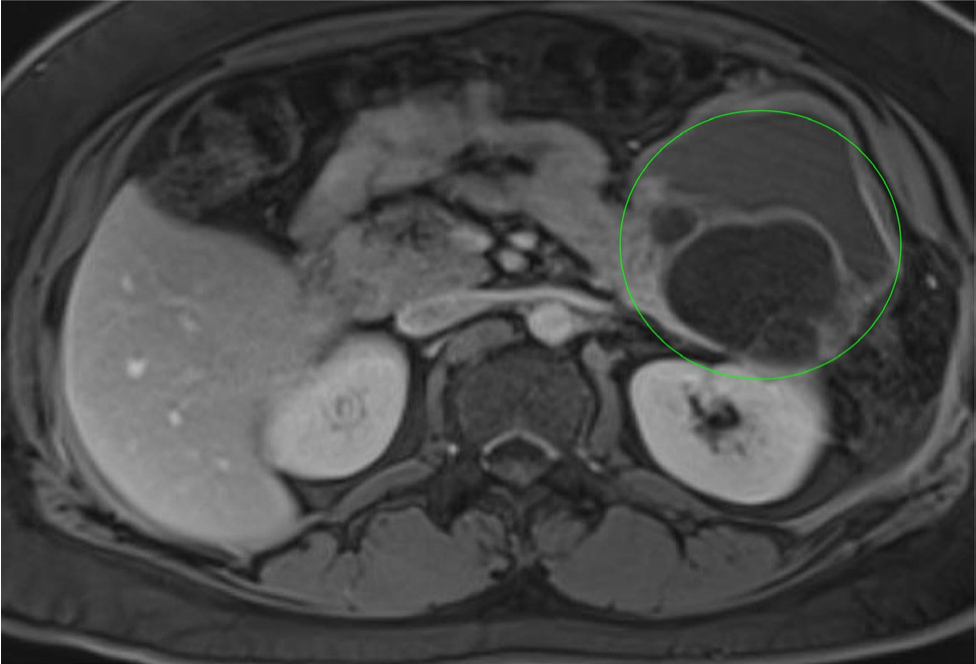
Abdominal magnetic resonance imaging/magnetic resonance cholangiopancreatography: abdominal imaging showing an 8.4 × 12.8 × 16 cm mass abutting the pancreas with moderate volume ascites.

**Figure 2.**
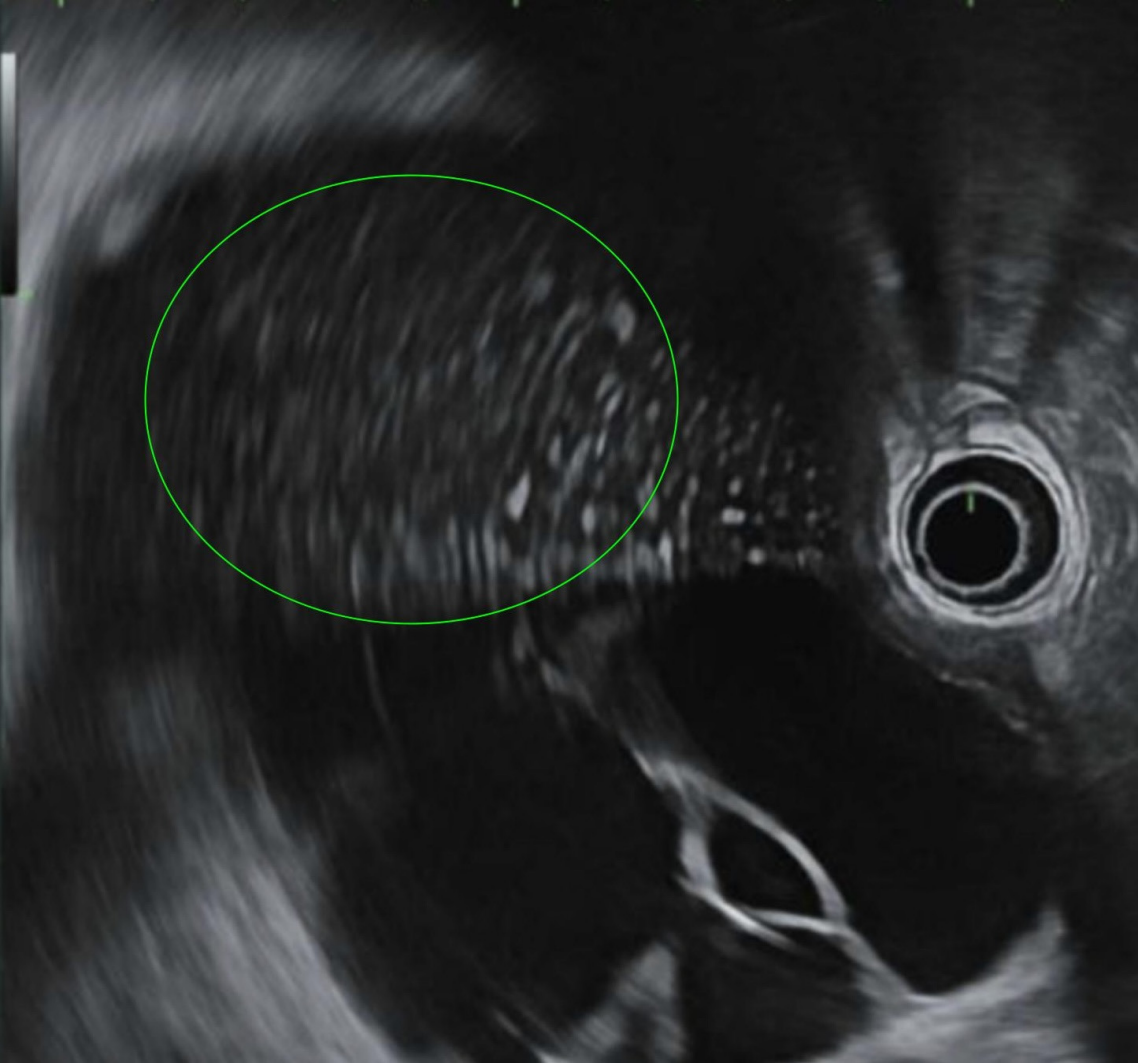
Endoscopic ultrasound of pancreatic cystic mass: EUS showing a 12 cm complex cystic mass encompassing the distal pancreas. EUS = endoscopic ultrasound.

**Figure 3.**
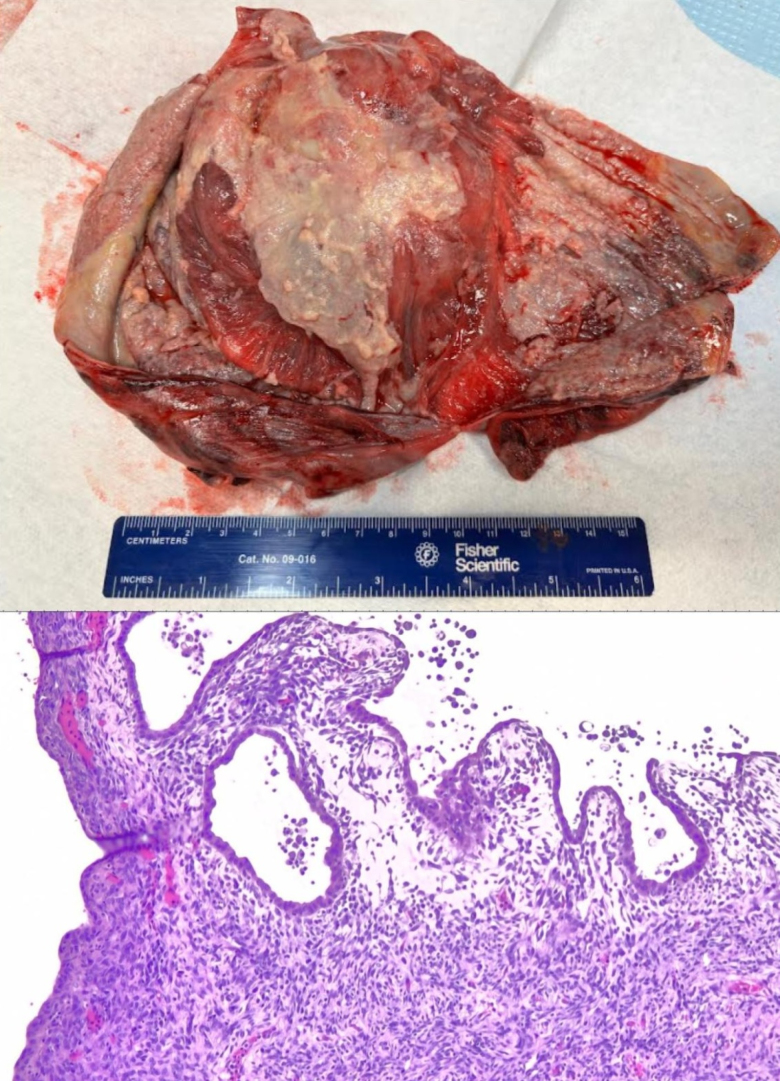
Mucinous cystic neoplasm: (A) Gross pathological specimen of the 14 × 11 × 7 cm multilocular cystic mass with a smooth outer surface, serous fluid, and a soft cystic wall measuring 5 cm from the surgical resection. (B) Hematoxylin and Eosin Stain at 200×, No evidence of high-grade dysplasia or invasive carcinoma, Margin of resection negative for tumor; consistent with a noninvasive low-grade MCN. MCN = mucinous cystic neoplasm.

A 24-year-old female with a past medical history of a pancreatic tail cystic mass presented with acute on chronic abdominal pain, nausea, and vomiting. She endorsed epigastric pain for the past year. Her cystic mass was identified in January 2024 on imaging, measuring 11.7 × 10.2 × 8.1 cm. Cytology from endoscopic ultrasound (EUS) performed at that time was non-diagnostic. On exam, she had diffuse abdominal tenderness. Labs were significant for a mild leukocytosis, microcytic anemia, elevated lipase of 385 U/L, and carbohydrate antigen 19-9 of 310 U/mL with normal CEA. Abdominal magnetic resonance imaging with magnetic resonance cholangiopancreatography showed a complex cystic mass measuring 8.4 × 12.8 × 16 cm abutting the pancreas with moderate volume ascites (Fig. 1). EUS with fine needle biopsy (FNB) was performed, showing a 12 cm complex cystic mass encompassing the distal pancreas (Fig. 2). Cystic fluid analysis showed elevated CEA of 42 ng/mL and amylase of 202,080 U/L. Molecular testing of cystic fluid using the PancraGen panel was negative for KRAS and GNAS mutations, loss of heterogeneity in tumor suppressor genes, or elevation in cystic DNA content. Based on this molecular testing, it was estimated that her lesion had a 97% probability of being benign over the next 3 years. Pathology showed a 14 × 11 × 7 cm multilocular, cystic mass with a smooth outside surface, serous fluid, and a soft cystic wall measuring 5 cm consistent with a noninvasive, low-grade MCN. There was no evidence of high-grade dysplasia or invasive carcinoma found, and the margin of resection was negative for tumor (Fig. 3). Post-EUS, the patient had intermittent fevers and was placed on piperacillin and tazobactam due to Aerococcus viridans growth in the cystic fluid. Given the patient’s significant symptom burden and the size of the cyst, she underwent a distal pancreatectomy with splenectomy, as the cystic mass was adherent to the splenic vessels at the splenic hilum, precluding a spleen-sparing procedure. She had an uncomplicated post-operative course and is following up with her outpatient providers post-resection of her MCN.

## 3. Discussion

A large benign MCN in a young female constitutes a rare case not only due to the early age of onset but also the exceptional size of the mass. Variation of cyst size among the various imaging modalities likely represented interval growth in some dimensions of the cyst as well as the inherent differences in image capacity/quality between the various measurement techniques. However, regardless of the variation in mass size between images, the lesion was large (far >3 cm) in all techniques. This case highlights that MCN should be considered in younger patients despite classically occurring in an older demographic.^[2,3,5,6]^ MCNs > 3 cm are at a higher risk of malignancy; however, despite their large size, they can be benign, as seen here.^[3,4]^ This indicates that size is a relative, and not absolute, risk factor.

It is rare for such a large MCN to be benign. However, the lack of other concerning radiographic findings such as enhancing mural nodules, pancreatic ductal dilation or peripheral calcifications increases the pretest likelihood of a benign process. Although KRAS/GNAS point mutations and loss of heterogeneity mutations are classically found in mucinous processes such as MCN, they were absent in molecular testing of this sample. KRAS mutations occur in both intraductal papillary mucinous neoplasm (IPMNs) and MCNs, with variable sensitivity.^[3,4]^ However, GNAS mutations are characteristic of IPMNs and very uncommonly seen in MCNs.^[7]^

Given that pathology confirmed the diagnosis of MCN, the lack of these molecular markers speaks to the benign nature of this patient’s cyst. A lack of somatic driver mutations, such as KRAS or TP53, is correlated with benign lesions irrespective of cyst size.^[7–9]^ Moreover, mucinous cysts typically have elevated cystic fluid CEA. A cutoff of 192 ng/mL has a specificity of 88.6% and sensitivity of 60.4%.^[10]^ Therefore, cases of MCN with low CEA can be missed.^[10,11]^ Cytology from EUS FNB cystic fluid analysis has a similar sensitivity and specificity for differentiating pancreatic cystic lesions.^[12]^ However, genetic testing for point mutations such as KRAS/GNAS can greatly increase sensitivity to 61 to 71% and specificity to 99% for differentiating between mucinous and non-mucinous pancreatic cysts.^[7]^

Differentiating MCN from other pancreatic cystic lesions, such as IPMN, serous cystadenoma, and pseudocyst, is important for management. Classical cyst fluid analysis for MCNs shows elevated CEA and low amylase, due to the absence of pancreatic ductal communication.^[13]^ In contrast, IPMNs and pseudocysts typically demonstrate high amylase due to ductal communication or pancreatitis.^[13]^ Serous cystadenomas have microcystic morphology, are low in cyst fluid CEA, and lack mucin production.^[13]^ In this case, the patient’s cyst fluid revealed a CEA of 42 ng/mL – below the 192 ng/mL cutoff used to differentiate mucinous from non-mucinous cysts – and a markedly elevated amylase of 202,080 U/L, creating a clear biomarker paradox.^[14]^ There are several possibilities to explain this discordance. One potential explanation is that sampling error or cyst heterogeneity produced non-representative fluid from a non-mucinous portion of the lesion. Alternatively, there could be partial ductal communication or fistulization. While rare, it is possible for high amylase levels to be present in MCNs if there is communication with the pancreatic duct, a nuance recognized in current guidelines from the American Gastroenterological Association and the European Society of Gastrointestinal Endoscopy.^[1,15,16]^ A mixed lesion – containing both MCN and IPMN components – has been described and could also account for this paradox, especially considering the patient’s chronic abdominal pain with nausea and vomiting.^[17]^

Definitive guidelines on the management of MCNs are lacking in the literature. The 2 pillars of management remain monitoring or surgical removal. Monitoring is employed for low-risk, asymptomatic cysts, while surgical removal is utilized for masses with higher risk features or significant symptom burden.^[1–3,15]^ A 2018 European Study Group guideline on PCNs such as MCN recommends conservative monitoring for asymptomatic masses > 40 mm without enhancing nodules and without other radiographic features concerning for malignancy.^[1]^ Lesions with high-risk features on imaging are treated with a pancreatectomy with the addition of splenectomy depending on the mass’s location and involvement of surrounding structures.^[1,3]^ Distinguishing between these 2 groups can be difficult based on image findings alone. Elevations in serum markers such as CEA and CA-19-9 have a strong positive predictive value for malignancy or a pre-malignancy. Specifically, a CEA >400 ng/mL has a strong association with malignancy in MCN.^[18]^ In addition to serum markers, EUS with FNB can serve as a vital tool in distinguishing the degree of cellular atypia and future risk of malignancy, allowing for more informed risk stratification.^[3,19]^ Thus, EUS, as employed in this case, provided vital information in guiding our patient’s clinical course.

This case also highlights the unique considerations which must be addressed in a young female, with a presumed long life expectancy, undergoing distal pancreatectomy and splenectomy. Young patients such as ours face lifelong implications of splenectomy, most notably increased susceptibility to infection with encapsulated organisms. Thrombosis and cancer are recognized as potential adverse outcomes of splenectomy as well.^[20]^ Strict adherence to vaccination protocols and consideration of long-term antibiotic prophylaxis is necessary as young patients have more years at risk for post-splenectomy infections.

Young females undergoing distal pancreatectomy and splenectomy also face increased risk in future pregnancies such as higher rates of preterm delivery, severe preeclampsia, cesarean delivery, and infectious complications.^[21]^ These increased risks highlight the importance of spleen preservation when it is feasible. This case was not amenable to spleen-sparing distal pancreatectomy given the adherence of the cystic mass to the splenic vessels at the splenic hilum.

Resection of noninvasive MCN, regardless of cellular atypia, is curative.^[3,22]^ Invasive MCN has a more variable illness course after resection. Early detection of MCN is crucial in monitoring and preventing progression to malignancy.^[1–3,6]^ Thus, even in patient populations outside of the typical demographic, MCN should be considered as the cause of a PCN. EUS remains a vital tool in the diagnosis and stratification of MCNs and PCNs as a whole.

## 4. Conclusion

We have illustrated that even an exceptionally large MCN can be benign. While cyst size is an important consideration in surgical decision-making, it should not be viewed in isolation. Comprehensive assessment, including imaging, cyst fluid, and molecular data, is necessary to accurately risk stratify and individualize patient care.

## Author contributions

**Conceptualization:** Ethan Burg, Gabrielle K. Sharbin, Max E. Edeson, Davood K. Hosseini, Hongfa Zhu, Rosario Ligresti.

**Data curation:** Gabrielle K. Sharbin, Max E. Edeson, Davood K. Hosseini, Hongfa Zhu, Rosario Ligresti.

**Investigation:** Davood K. Hosseini.

**Methodology:** Davood K. Hosseini.

**Project administration:** Davood K. Hosseini.

**Software:** Davood K. Hosseini.

**Supervision:** Davood K. Hosseini.

**Writing – original draft:** Ethan Burg, Gabrielle K. Sharbin, Max E. Edeson, Hossein Rabiei Samani, Hanieh K. Hosseini, Davood K. Hosseini, Shil Patel, Jonathan D. Weinberger, Hongfa Zhu, Rosario Ligresti.

**Writing – review & editing:** Davood K. Hosseini.
